# 334. Efficacy, Safety, and Completion of Modified Short-Course Rifapentine and Isoniazid for Latent Tuberculosis Infection in High-Risk Patients with Rheumatic Disease: A Multicenter Randomized Controlled Trial

**DOI:** 10.1093/ofid/ofaf695.117

**Published:** 2026-01-11

**Authors:** lifan zhang, yujie he, wenwen wang, lijun wu, xiaoxia zuo, sheng chen, yanping zhao, ping zhu, hongbin li, xiaoqing liu

**Affiliations:** Peking Union Medical College Hospital,, Beijing, Beijing, China; The First Affiliated Hospital of Zhengzhou University, Zhengzhou, Hainan, China; The Second Affiliated Hospital of Zhejiang University School of Medicine, Hangzhou, Zhejiang, China; People’s Hospital of Xinjiang Uygur Autonomous Region, Urumchi, Xinjiang, China; Xiangya Hospital, Central South University, Changsha, Hunan, China; Renji Hospital, School of Medicine, Shanghai Jiao Tong University, Shanghai, Shanghai, China; The First Affiliated Hospital of Harbin Medical University, Harbin, Heilongjiang, China; Xijing Hospital, Fourth Military Medical University, Xi’an, Shanxi, China; The Affiliated Hospital of Inner Mongolia Medical University, Hohhot, Nei Mongol, China; Peking Union Medical College Hospital, Beijing, Beijing, China

## Abstract

**Background:**

Patients with rheumatic diseases (RDs) have a high risk of latent tuberculosis infection (LTBI) reactivation. However, research on tuberculosis preventive treatment (TPT) in this specific patient group remains limited. Additionally, the safety of the WHO - recommended 3 - month regimen of weekly rifapentine (RFT) plus isoniazid (INH) (3HP) has raised concerns among the Chinese population. This study aims to evaluate the efficacy, safety, and treatment completion of a modified 3HP regimen to a 9 - month INH monotherapy regimen in this vulnerable population.

**Figure:**
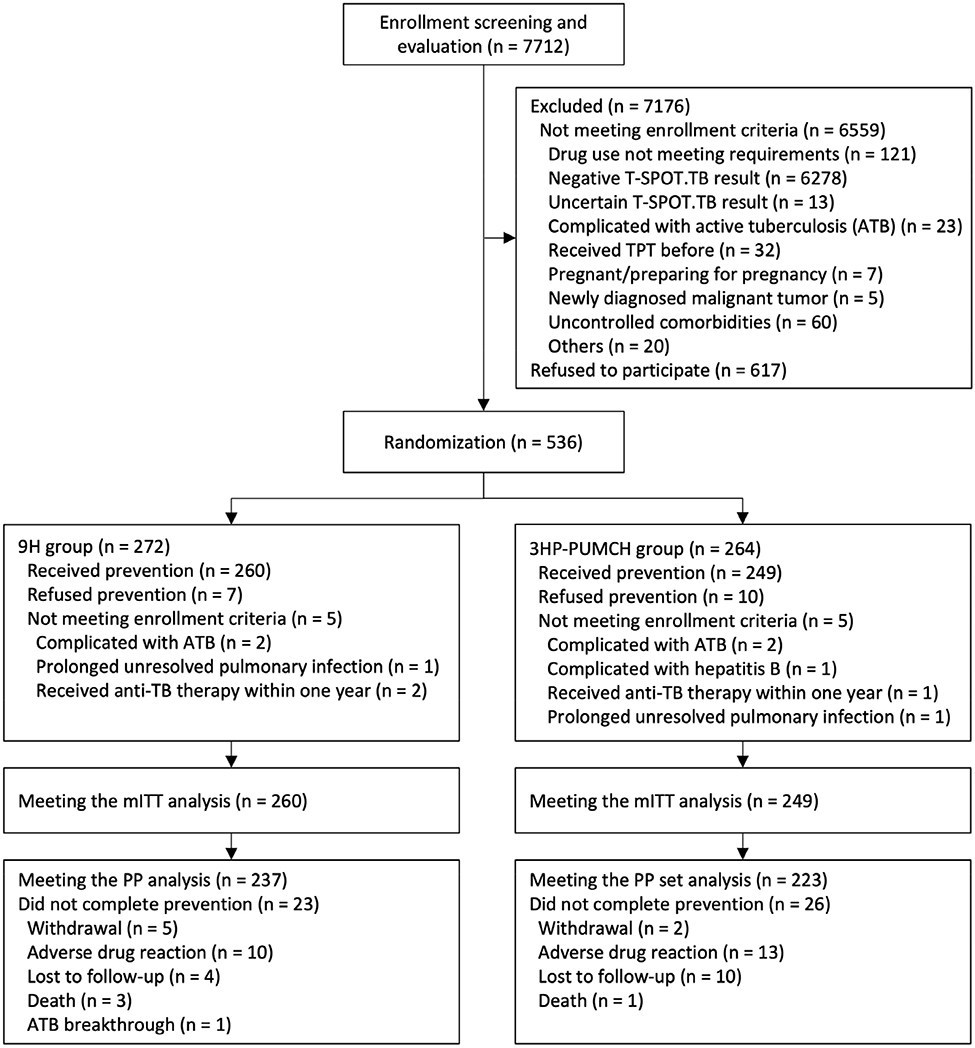
Flowchart of study participant intervention and follow-up.

**Figure:**
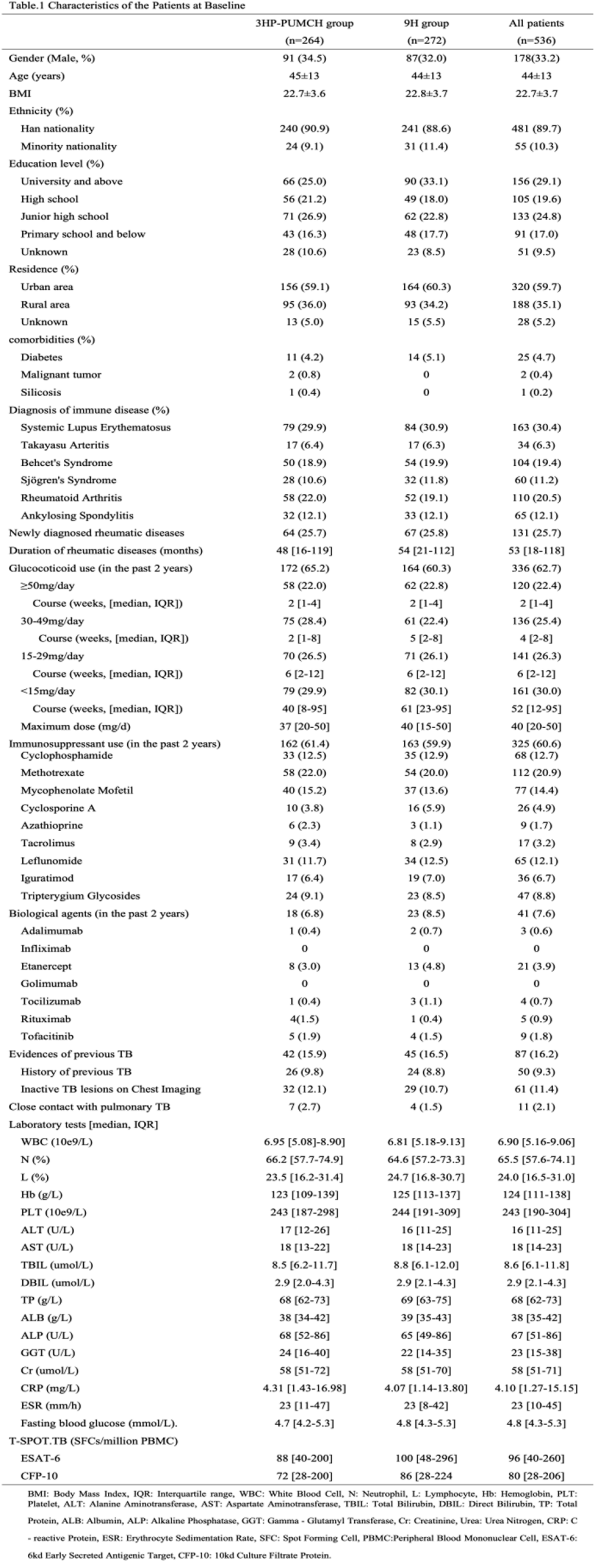

**Methods:**

We conducted a multicenter, open-label, randomized noninferiority trial comparing a modified 3-month regimens of twice weekly RFT at 450mg plus daily INH at a maximum dose of 300mg (3HP-PUMCH) versus 9-month daily isoniazid at a maximum dose of 300mg (9H) in patients with RDs. Subjects were enrolled from 9 tertiary hospitals across China and followed for 2 years. The primary endpoint was confirmed active TB (ATB), with a noninferiority margin of 1.4%.

**Figure:**
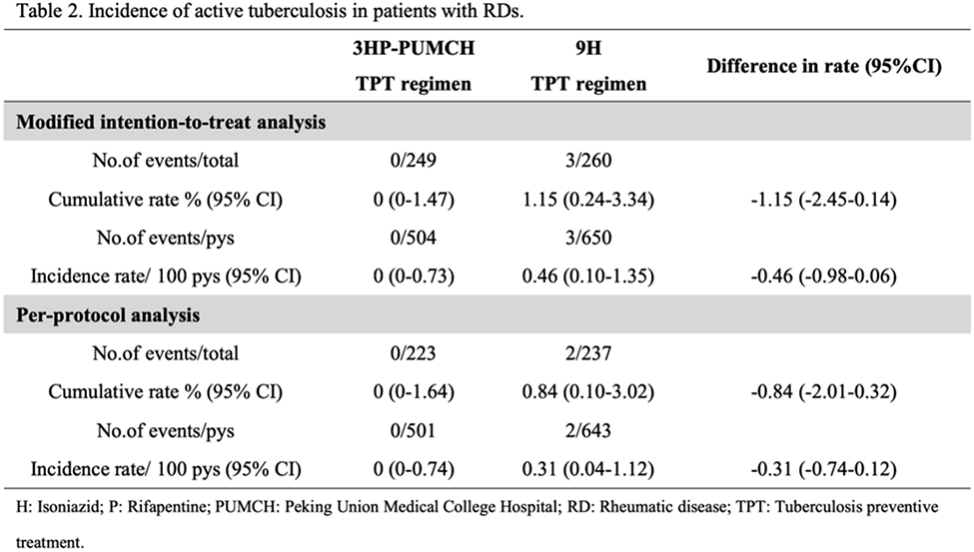

**Figure:**
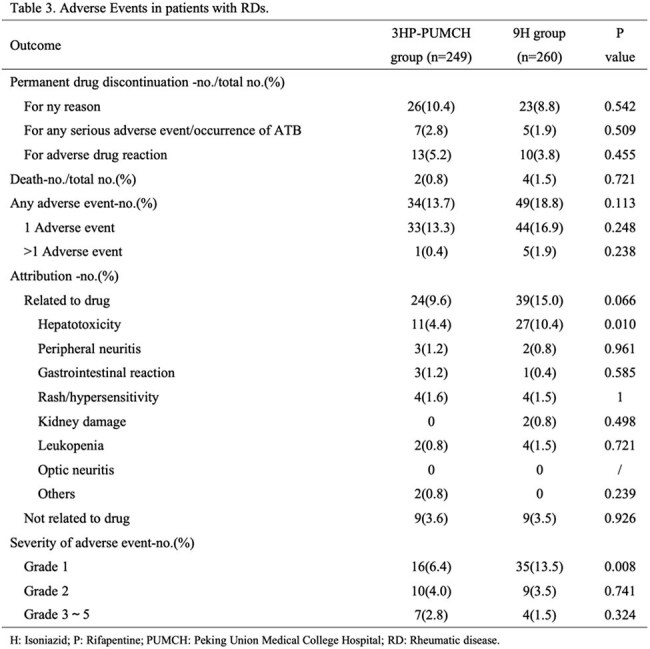

**Results:**

A total of 536 patients with RDs were enrolled. In the modified intention-to-treat analysis, no cases of ATB occurred among 249 RDs patients in the 3HP-PUMCH group, with a cumulative rate of 0.00% (95% confidence interval [CI] 0.00 - 1.47), while 3 cases were observed among 260 patients in the 9H group, with a cumulative rate of 1.15% (95% CI 0.24 - 3.34), and the rate difference was -1.15% (95% CI -2.4 - 0.14). Rates of drug discontinuation due to serious adverse events or the occurrence of ATB in the 3HP-PUMCH group and the 9H group were 2.8% and 1.9% respectively (p=0.509), rates of investigator-assessed preventive drugs-related adverse reactions were 9.6% and 15.0% respectively (p = 0.066), with the rates of hepatotoxicity related to preventive drugs were 4.4% and 10.4% respectively (p = 0.010). Treatment completion rates were 89.6% in the 3HP-PUMCH group and 91.2% in the 9H group (p=0.542).

**Conclusion:**

The 3HP-PUMCH was as effective as the 9H in preventing ATB and demonstrated a favorable safety profile and high completion in LTBI patients with high-risk RDs. Moreover, this novel TPT regimen might be more suitable for patients with underlying diseases and those on concomitant medications, potentially offering a more practical and manageable option in complex clinical scenarios.

**Disclosures:**

All Authors: No reported disclosures

